# Barriers and facilitators of adherence to treatment among women with vulvovaginal candidiasis: a qualitative study

**DOI:** 10.1186/s40001-022-00938-y

**Published:** 2022-12-21

**Authors:** Maryam Erfaninejad, Arash Salahshouri, Nasrin Amirrajab

**Affiliations:** 1 Medical Mycology, Student Research Committee, Ahvaz Jundishapur University of Medical Sciences, Ahvaz, Iran; 2 Department of Health Education & Promotion, School of Health, Ahvaz Jundishapur University of Medical Science, Ahvaz, Iran; 3 Department of Laboratory Sciences, School of Allied Medical Sciences, Ahvaz Jundishapur University of Medical Sciences, Ahvaz, Iran

**Keywords:** Recurrent vulvovaginal candidiasis, Qualitative research, Treatment failure

## Abstract

**Background:**

Non-adherence of patients with vulvovaginal candidiasis (VVC) to treatment recommendations leads to treatment failure and recurrence of infection. Therefore, this qualitative study was conducted to identify barriers and facilitators of observance of treatment among women afflicted with vulvovaginal candidiasis.

**Methods:**

This qualitative study was conducted through 26 in-depth unstructured interviews with 24 patients and 2 gynecologists using purposeful sampling with maximum variation in Ahvaz, southwest Iran. Interviews were conducted in person at health centers and the gynecologist’s offices. MAXQDA 10 software and conventional content analysis were used for data analysis.

**Results:**

The findings showed barriers and facilitator factors of adherence to treatment in women with VVC. Some of these factors lead to an increase in adherence to treatment, and others play the role of hindering factors. These factors were classified into two main categories: patients’ beliefs and patients’ fears and concerns.

**Conclusion:**

The results of this study showed that many of the behaviors of patients from the acceptance of the diagnosis process to treatment are rooted in the patient's beliefs and fears. Therefore, it seems necessary to design and carry out interventions based on the findings of this study, which can be used in the development of appropriate solutions, treatment guidelines, and adopting a policy for treatment adherence.

## Background

Vulvovaginal candidiasis (VVC) is the second most common cause of genital infection among women of reproductive age [1]. VVC is typically characterized by vaginal soreness, itching, dyspareunia, external dysuria, discomfort during sex, and abnormal vaginal discharge [2]. An estimated 75% of women experience VVC at least once during reproductive age [3, 4]. It has been found that 40–45% of women suffer from two or more episodes within one year, which is defined as recurrent vulvovaginal candidiasis (RVVC) [5]. According to reports, approximately 140 million women are experiencing RVVC annually [3]. The cause of infection is *Candida* yeast, especially *Candida albicans* species, which is part of normal vaginal microflora and becomes a robust opportunistic fungal pathogen under various conditions [6]. Some of the risk factors associated with VVC development include an estrogen-rich environment, an immunocompromised state, increased vaginal glycogen levels, uncontrolled diabetes, genetics, frequency of sexual activity, hygiene habits, dysbiosis of the vaginal microbiome, atopy, and idiopathic origins [1, 6–8]. Intravaginal and oral azole drugs are being currently prescribed for the treatment of VVC [9–11]. Topical formulations of azoles, clotrimazole, miconazole, and tioconazole, in particular, are available over the counter (OTC) [12]. However, misuse of medications or incomplete treatment and non-adherence to treatment recommendations will directly or indirectly lead to treatment failure [8–10].

VVC not only has a wide range of adverse effects on the physical, psychological, and social dimensions of women's life, but also affects their quality of life. The symptoms and predisposing factors of VVC are well known. However, significant cases of this infection are idiopathic [2]. Under such circumstances, studying the factors that determine the success of patients' treatment has become very important. Because most of the studies have been conducted to identify the pathophysiology, epidemiology, drug effectiveness, and drug resistance of VVC [9–11, 13–15] and to the best of our knowledge, few scientific efforts have been made to investigate the determinants of patients' treatment behaviors [16].

According to previous studies, there is a high prevalence of VVC in Khuzestan (39.79%) [13], and the researchers of the present study noticed a series of behaviors and beliefs during their past experiences about women suffering from VCC, which led to patients' non-adherence to treatment. Also, considering the complexity of the causes of VVC infection, there are likely other hidden reasons that have not been evaluated. Therefore, in this study, a qualitative method was used to identify barriers and facilitators of adherence to treatment among women with vulvovaginal candidiasis.

## Materials and methods

### Settings

The study settings were clinics, healthcare centers, and gynecologists’ offices in Ahvaz, Iran, to identify barriers and facilitators in the way of adherence to treatment among women with vulvovaginal candidiasis.

### Study design

The present study is a qualitative study using in-depth unstructured face-to-face interviews with women with a history of VVC, and in some cases by phone call to complete the interview to explain the factors involved in treatment failure and recurrence of VVC infection. For this purpose, an inductive approach was adopted, and data analysis was performed using content analysis [17].

### Participants and sampling

Purposeful sampling with maximum variation was used to select 26 female participants, including 24 patients and two gynecologists [18]. In Iran, women receive the required health services by visiting healthcare centers or gynecologists for free or for a fee, respectively. One of these services is the Pap smear test which is used to check for cervical cancer or certain vaginal and uterine infections [19, 20]. Any fungal infection is detected through this test. Therefore, participants of this study were women who met the following criteria: confirmation of VVC by Pap test, history of treatment failure or in other words RVVC,^1^ and willingness to participate in the study. The unwillingness of the participant to continue cooperation, and non-cooperation in expressing experiences for various reasons such as shame and unavailability of the Pap test result caused the participant to be excluded from the study.

Initial explanations were presented by the gynecologist or health care provider during the patient's visit who was introduced to us in case of consent. In the next step, researchers tried to gain the participants' satisfaction by explaining the goals of the project to them and that their experiences can have a positive effect on the discovery of unknowns affecting the treatment process. Due to the ethnic diversity in the city of Ahvaz, participants from different ethnicities, with diverse age ranges (18–50 years) and various sexual functions were selected. Each participant was fully briefed on the research method and objectives in plain and clear language and was assured of the confidentiality of the information obtained from them. In-depth unstructured interviews were conducted separately for each participant in a relaxed atmosphere after they had signed the written informed consent form [21]. To obtain more comprehensive and in-depth data, other informers were also interviewed, including two gynecologists with at least 3 years of experience in teaching and treating patients with VVC. Because all participants were sexually active, two sexually inactive patients were selected and interviewed to ensure maximum participant diversity in terms of sexual function. Unstructured in-depth interviews continued until data saturation, i.e., when no findings or new codes were obtained from the participants' interviews. After 26 interviews, data saturation was achieved. A total of 29 people were invited to participate in this study. Three women were excluded for inability to speak Persian, one for time constraints, and another for her husband’s denial of consent.

### Data collection

The method of data collection in this study was through unstructured in-depth interviews and field notes. The interviews and data collection process was conducted from September 2020 to January 2021. The first (ME) and second (AS) authors were responsible for conducting the interviews as trained, experienced interviewers who did not comment and only took notes. The interview started with asking demographic questions (such as age, education, number of family members, marital status, etc.) to get to know each other better and create a friendly environment. The interview then proceeded in an unstructured format with a general question, which asked participants to describe their feelings and experiences related to VVC infection. Next, according to the participant's explanations and the interviewer's experiences, if the participant's statements were ambiguous, probing questions such as "Please explain what you said…", and "Can you explain your answer more?", "Do you mean…." were inquired to express all their experiences? At the end of each interview, the participants were requested to share any information they felt was important but not covered in the interview. Each interview lasted from 20 to 60 min. During the interview, active listening techniques were used by watching out for body language cues. Taking notes of the discussions by the research assistant helped to continue the in-depth interview about each topic away from bias. In the end, the notes were reviewed by the participants to confirm the truth of the statements. Also, immediately after each interview, field notes were recorded as another way to collect data and assess participants' nonverbal behaviors. Before the interview, the tape recorder was activated with the knowledge and consent of the interviewees, and they had the opportunity to share their personal opinions and experiences about VVC. At the end of each interview, the recorded audio was carefully listened to several times and transcribed verbatim, and the transcription was compared with the audio recording to avoid discrepancies. Finally, 26 interviews (including 24 patients and 2 gynecologists) were conducted in person in quiet rooms without the presence of any responsible person from clinics (4 interviews), healthcare centers (12 interviews), and gynecologists’ offices (10 interviews).

### Data analysis method

Data were reviewed repeatedly to inform the purposive sampling strategy following the principle of theoretical saturation [21]. Then, interview transcriptions and field notes were imported into qualitative data analysis software (MAXQDA 10) and were subjected to a content analysis model according to the steps defined by Braun and Clarke [22]. For this purpose, from the list of keywords and phrases, an initial coding framework was created in MAXQDA 10 software. The first-level codes were classified according to their differences and similarities. Subsequently, second-level codes were generated by naming each category, repeating the categorization, combining similar codes, and adding new emerging codes, which led to the extraction of the main themes. The inductive method was meant to classify the codes and the deductive approach was used to compare the category in the opposite direction. After analyzing all the data and agreeing on the categories, each group was evaluated independently for data saturation. Data saturation refers to the point in the research where no new information and perception is discerned in the data analysis. As the same items were reviewed over and over again, it became clear that data saturation had occurred.

### Scientific trustworthiness of the results

**Fig. 1:**
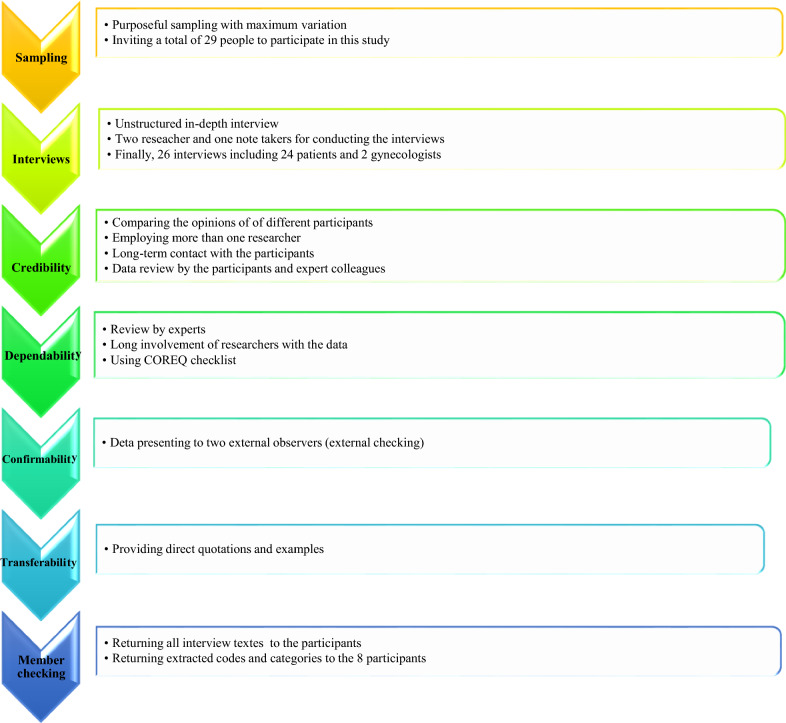
Summary of data analysis

The purpose of scientific accuracy in a qualitative study is to correctly express the real experiences of the participants [23]. The reliability of the study was evaluated based on four criteria determined by Lincoln and Guba [24]: (a) to assess the credibility of the data, the opinions of patients and gynecologists with different perspectives were compared to ensure the triangulation of the data source. In addition, the study was designed to include more than one researcher. Long-term contact with the participants, appropriate interaction with them, and data review by the participants and expert colleagues increased the credibility of the data; (b) to increase the dependability of data, actions such as step-by-step repetition, data collection, and analysis, review by experts, long-term involvement of researchers with the data and immersion in the data were carried out. During the survey, the authors used consolidated criteria for reporting qualitative research (COREQ: 32-item checklist) to ensure accuracy in the present study [25]. (c) To obtain confirmability, data were presented to two external observers (external checking). (d) Also, providing direct quotations and examples increased the transferability criterion. To increase the content validity, all texts from the interviews were returned to the 26 participants. In addition, extracted codes and categories were returned to 8 participants to confirm and comment (member checking). The data analysis process is summarized in Fig. 1.

## Results

This study is descriptive qualitative research that was conducted to explain the factors related to the recurrence of VVC infection and treatment failure in Khuzestan province. The demographic information collected from the participants before each interview is shown in Table 1. Data saturation was obtained after 26 interviews. Analysis of data related to infection recurrence and treatment failure in women with VVC led to the extraction of factors associated with treatment failure and disease recurrence. In this study, two main categories, namely patients’ beliefs, and patients’ fears and concerns are discussed along with their subcategories (Table 2).

**Table 1:** Characteristics of 24 interviewed participants

Variable	N (%)
Education	
Elementary and middle school	10 (41.7)
Associate degree	3 (12.5)
Bachelor’s degree	7 (29.1)
Master’s degree	3 (12.5)
Doctor of Philosophy	1 (4.2)
Job-status	
Housewife	14 (58.3)
Employed	10 (41.7)
Sexual status	
Sexually inactive	2 (8.3)
A sexual partner	19 (79.2)
More than one sexual partner	3 (12.5)
Candidal vaginitis status	
Less than 1 year	11 (45.8)
1 to 2 years	4 (16.7)
3 to 5 years	5 (20.8)
More than 5 years	4 (16.7)

**Table 2:** The codes, subcategories, and categories of the factors related to infection recurrence and treatment failure in women with VVC

Category	Subcategory	Code
Patients’ beliefs	Beliefs related to human genetics and physiology	The patient believes that the infection is part of the menstrual cycle
		Self-medication because of the belief that female hormones cause infection
		Withdrawal from medication due to the belief that infection is treated spontaneously after marriage
		Ignoring treatment recommendations due to the belief that genetics affects infection
	Beliefs related to the patient's physical and mental condition	Ignoring treatment recommendations due to the patient's depressive condition
		Quitting medication due to impatience in medication adherence
		Lack of treatment due to the stress caused by family problems
		Ignoring the treatment process because of the belief that work stress plays a role in the recurrence of infection
		Belief in the effect of being overweight and obese on the recurrence of vaginal infections
		Belief in a higher probability of recurrence of infection in women with hyperhidrosis
	Beliefs related to the effectiveness of drugs	The inefficiency of routine drugs in the treatment of fungal infections
		Patients’ belief in the greater impact of pills and ampoules than traditional treatments
		The patients’ belief that pills are more effective than the topical cream
		Disbelief in prescription drugs in controlling the symptoms of the disease
	Beliefs related to individual health	Belief in the association of daily pad use with recurrent infections
		Belief in the relationship between disease recurrence and vaginal gel application
		Patients’ belief in the effect of late replacement of sanitary pads during menstruation on the development of infection
		Belief in the association of obsessive behaviors with individual health and recurrent infections
	Beliefs related to intercourse and contraceptives	Belief in the effect of having sex in RVVC
		Belief in the recurrence of infection following unprotected sex (without condoms)
		Not taking medication due to belief in the effect of IUD on disease recurrence
		Belief in the effect of birth control pills on infection
Patients’ fears and concerns	Fear of drug side effects	Not taking the medication completely out of fear of feeling nauseous
		Not taking medication out of fear of unknown side effects of using chemical drugs
		Failure to take the drug completely due to fear of re-experiencing an allergic reaction
		Not taking medication due to fear of side effects
		Not taking medication for fear of adverse effects on the liver
	Fear of not recovering	Proper and timely use of medication for fear of recurrence of infection
		Accurate and timely use of medication out of fear of not recovering
	Concern about the possibility of needing advanced treatments in the future	Careful use of the drug out of fear of being prescribed cryotherapy
	The concern of being examined	Refraining from being examined due to the concern of vaginal injury during the examination
		Fear of getting infected by being examined by a specialist
	Panic caused by the COVID-19 pandemic	Reluctance to be examined out of fear of contracting the coronavirus
		Not seeing a doctor out of fear of getting coronavirus

### Patient's beliefs

According to the results, 21 participants expressed their beliefs as one of the main reasons for treatment failure or infection recurrence. Based on the data analysis, participants provided five beliefs related to this issue as follows.

#### Beliefs related to human genetics and physiology

Physiological factors here refer to hormonal changes and the menstrual cycle in women, which participants believed could contribute to infection recurrence or improvement spontaneously. A 42-year-old patient with a 2-year history of RVVC commented on the effect of the menstrual cycle on the recurrence of infection:

"In my opinion, the infection has become part of my menstrual cycle because the infection recurs in each menstrual cycle and therefore the treatment has no effect on me”.

Another factor that led the participants to arbitrary treatment is the belief in the effect of hormones on the recurrence of infection. Some participants believed that hormonal changes in the body cause a recurrence of the infection every month, so it does not make sense to see a doctor and take full medication for each infection every month. One participant remarked:

“I believe that the infection is compatible with my hormonal changes, and as a result, it is useless for me to be concerned about it and use the antifungal vaginal cream (clotrimazole 1%).” (35-year-old woman with 5 years of history of RVVC).

Some participants considered genetics to be an effective factor in the recurrence of infection and believed that genetics is an important factor in the development of VVC and that recurrence of the infection is inevitable despite individual health and follow-up treatment. Another participant revealed:

“I do not like to see a doctor because my mother, despite being 50 years old, still suffers from this infection and medical treatments have not been effective. I believe that genetics is very influential, and I think my genes are the same as my mother’s, so treatment is useless" (42-year-old woman with 18 years of RVVC history).

#### Beliefs related to the patient's physical and mental condition

According to the participants, the beliefs related to the patient's mental condition can be an effective factor in ignoring treatment recommendations and drug withdrawal. Several participants cited depression, impatience, and stress associated with family and work as major barriers to pursuing treatment. One sexually inactive participant, aged 38 with a 1-year history of RVVC, argued:

“My work conditions are very stressful and these tensions and work stress cause my infection to constantly recur and the treatment to be ineffective for me”.

Regarding beliefs that hinder treatment, another participant stated:

“I’m always preoccupied with the everyday problems I have, and I do not have the patience to take care of myself at all” (38-year-old woman with a 3-month history of VVC).

Other participants believed that their physical condition could affect the treatment of the infection. One patient said:

“I can’t stop eating, so the wrinkles in my genital area which are caused by obesity and subsequent sweating, do not let my infection be treated” (a 42-year-old woman with a history of 2 years of RVVC).

#### Beliefs related to the effectiveness of drugs

According to our results, beliefs related to the effectiveness of drugs are a strong barrier to pursuing treatment and adherence to medications. A 33-year-old participant, who developed VVC 1–2 times a year and whose recent infection had failed treatment for 3 months, claimed:

“I believe the medications prescribed to treat my infection are not strong enough. So, I don't insist on taking my medicine either”.

#### Beliefs related to individual health

Data analysis showed that beliefs related to individual health affect the motivation of some participants to continue treatment or adhere to prescribed medications. Some participants believed that repeated use of daily pads and vaginal gels, delays in changing sanitary pads, and obsession with individual health all contributed to the development or recurrence of infection. In this regard, a participant revealed:

“Vaginal gels do not meet the required standards and can cause a recurrence of the infection by irritating the vagina…and it causes me to get involved again.” (a 35-year-old woman with a history of three VVC cases in 5 years).

Also, a 38-year-old woman with a history of recurrence of the infection several times in one year explained:

“Obsessive and excessive use of detergents causes my infection to recur. So, the medicine is not effective”.

#### Beliefs related to intercourse and contraceptives

Another belief that participants discussed directly or indirectly were related to intercourse and the related topics. While many participants blamed intercourse, especially unprotected intercourse, for treatment failure or recurrence of infection, some believed that intercourse does not interfere with treatment, but contraceptives such as condoms and birth control pills, predispose the vagina to recurrence of infection or cause treatment failure. A 42-year-old participant asserted:

“I have had a fungal infection since I used IUD (for 18 years) and my infection has improved since I removed IUD (during the last month). So, IUD is the cause of infection.”

Another patient said: “I think condoms play a role in exacerbating the infection, and because my husband is not satisfied with using a condom, my infection keeps recurring.” (32-year-old participant with 8 years of history of VVC infection 1–2 times a year).

A 38-year-old patient with a 3-year history of RVVC infection stated: “I believe the cause of my recurrence of the infection is taking birth control pills. So, it is pointless to continue taking the drug”.

### Patient’s fears and concerns

Patients’ fears are and concerns influential factors that can lead them to pursue treatment or act as a strong barrier to taking medication and even seeing a doctor. This category involves the following five subcategories.

#### Fear of drug side effects

Fear of side effects of drugs was one of the major fears of patients that led them to refrain from taking the drug. A 34-year-old woman, who experienced failure of VVC treatment after marriage for several months, explained: “I never take the drug completely because I feel that the drug is a chemical that has more of a side effect than a positive effect and should be used in an emergency and only to relieve acute conditions.”

Two participants experienced severe allergic reactions while taking medications, and fear of the recurrence of such reactions led to their reluctance to take any drug. One of the participants said: “The gynecologist prescribed a medicine to treat VVC infection, but my body was not compatible with these drugs, and after taking these drugs, I got hospitalized. Eventually, I had to give up my medication” (33-year-old woman with a history of treatment failure within a period of three months).

#### Fear of not recovering

Sometimes the patient's fears act as a facilitator and motivator in pursuing treatment. Fear of untreated or recurrent infection caused some participants to be careful in the timely taking of the right medications. One patient remarked:

“I'm afraid that the infection will come back if I do not take the medicine on time. So, I paid attention to everything while taking the medicine” (34-year-old woman with a history of failure of VVC treatment for several months).

#### Concern about the possibility of needing advanced treatments in the future

Concerns about cryotherapy, due to non-adherence to treatment recommendations and as a result of worsening of infection, encouraged some patients to take drugs accurately and completely.

A 28-year-old participant who had a recurrent infection for six months after delivery said:

“The specialist intends to prescribe cryotherapy if the infection recurs ... and since I am afraid of cryotherapy, I am very careful in taking my medications”.

#### The concern of being examined

Data analysis showed that the concern of developing infection and damaging the vagina during the examination caused some patients not to see a specialist for treatment. Therefore, there was a time lag in receiving medications. A 27-year-old participant with an occasional history of VVC described her experience: “A while ago, I went to see the doctor for a pre-pregnancy check-up and examination, but I got VVC infected. After that bitter experience, I neither want to see a specialist nor allow a doctor to examine me”.

A gynecologist explained as follows: “The concern of examination causes treatment measures to be provided based on patient’s explanations, and this increases the possibility of prescribing the wrong medicine, and as a result, the patient returns to the doctor's office and expresses dissatisfaction with the recovery process”.

#### The panic caused by the COVID-19 pandemic

The fear and panic of the possibility of contracting the disease during the COVID-19 pandemic caused patients not to seek treatment after the end of the VVC treatment period, as a result, the treatment process was accompanied by challenges. One of the participants explained: “Although I have run out of medicine, I do not see a specialist at all for the panic of getting the coronavirus” (a 30-year-old patient with a history of self-medication for recurrent infections).

## Discussion

This qualitative study aimed to describe the experiences of women with VVC in terms of infection recurrence and treatment failure. A qualitative approach using unstructured in-depth interviews allowed participants to share their experiences with the authors on the reasons for treatment failure or recurrence of VVC infection, and this study reports part of their experience. Qualitative data analysis identified two categories of factors, namely patients’ beliefs and patients’ fears and concerns, along with their subcategories. Despite an extensive review of the literature, no similar study was observed to be conducted on this topic. The present findings pave the way for further studies and interventions by providing different categorizations and new definitions of concepts.

The word ‘belief’ is a term in social psychology that is related to psychological phenomena which lead to behavior building. In most interviews, the participants expressed negative beliefs about physiological and genetic factors, physical and mental conditions, the effectiveness of drugs, individual health, and intercourse and contraceptives as barriers to their adherence to and follow-up of treatment. Several participants cited physiological changes in their bodies during menstruation, including monthly hormonal changes, as the main cause of recurrent infections. Because people cannot change their genetic makeup or physiological factors, having such beliefs discourages patients from pursuing treatment because they consider the disease incurable. Under such circumstances where patients under the influence of negative beliefs find a complete cure unlikely, they try to control the infection only by relying on their knowledge. Since a significant portion of the patient's knowledge is probably rooted in pseudo-science or obtained from unreliable sources, this can lead to the extension or recurrence of infection instead of improving the condition.

Several participants cited their physical condition as the reason for the failed treatment of the infection. They believed that their physical conditions, such as obesity or excessive sweating, can lead to infections. Most of these participants stated that they had obtained information on this from specialists while referring to them for treatment of the infection, and the doctors, instead of referring the patient to the relevant specialist, asked the patient to control the infection and keep her body in the ideal condition. Therefore, the patient tried to solve her problem based on her knowledge. However, because these efforts did not conform to scientific principles, they failed, and these failures began to instill into them the belief that due to their physical condition, there is no cure.

Given the immense impact of the specialists’ recommendations on the formation of patients' beliefs, specialists should explain the problem, offer solutions, and give the patient sufficient motivation and confidence to pursue treatment. In the meantime, bad mental conditions such as boredom, depression, and stress caused by work or family conditions undoubtedly have a great impact on adherence to treatment. Therefore, the patient must have an acceptable level of mental and emotional preparation before starting the treatment process so that the treatment can be started with sufficient motivation and followed with the necessary care. This is a point that most doctors in Iran do not usually pay attention to, and they attribute failure in treatment follow-up to the patients’ ignorance of the doctor's advice.

Some participants believed that drugs do not work or that only certain types of drugs are effective for treatment. As a result of these beliefs, the patient's motivation to continue treatment, use the drug carefully, and complete the course of treatment is considerably reduced. A study by Bilardi et al. identified patient frustration and dissatisfaction with current treatment regimens for bacterial vaginosis as a factor in women's desire for self-medication and subsequent recurrence of infection. They found that most women failed to control their vaginal infections through self-help remedies or lifestyle modifications [26], which is consistent with our results.

Many participants tried to control the recurrence of the infection by modifying their health habits (for example, regular vaginal cleansing with different types of detergents and deodorants or using daily pads and changing pads at predetermined intervals). However, these efforts were either not always practical or gradually turned into obsessive behaviors and were performed out of moderation. Therefore, the patients believed that such a style of individual health was the cause of the recurrence of infection, but since they did not consider themselves capable of quitting obsessive behaviors, recurrence of the infection seemed inevitable to them. In addition to patients who attributed their obsession with individual health to failure in their treatment, several participants found the use of certain health products, such as women's vaginal cleansing gels or sanitary pads, to affect their infection.

Several participants also believed that delay in changing the sanitary pad during menstruation is an important factor in the onset of infection. However, many of these beliefs have no scientific basis. Therefore, participants' beliefs about individual health can be an effective barrier to pursuing treatment. Some of these behaviors are rooted in obsessive–compulsive disorder (OCD). OCD involves thoughts, images, and impulses that are constantly repeated in a person's mind and seem out of control, and come to mind against the patient’s will [27–29]. Researchers have shown that obsessive–compulsive individuals have a weaker immune system [30], and as a result, these obsessive–compulsive behaviors in vaginal cleansing can weaken a woman's local immune system in the vaginal area. To solve such a complex situation, prescribing medication alone is not the cure for the infection, and the patient must first be helped to control obsessive–compulsive disorder. Then the patient begins the VVC treatment process with appropriate mental and physical fitness.

Participants' beliefs about intercourse and contraceptives play an important role in motivating patients to seek treatment. Analysis of the data of the present study showed that contradictory beliefs about intercourse and contraceptives have been formed in the patient’s mind. On the one hand, some participants attributed infection recurrence or treatment failure to having sex during treatment, condom-free intercourse, taking birth control pills, and the use of IUDs, and subsequently refused to take medication and considered pursuing treatment futile. On the other hand, some had never been challenged on such topics and were surprised by the researchers' questions. This difference in participants' attitudes indicates a low level of information about the topic of this study, which is consistent with Khalesi et al.’s finding regarding the depth of participants' lack of knowledge about sexual issues [31]. Therefore, to change their beliefs, patients' knowledge and awareness about sexual intercourse, contraception, and sexually transmitted diseases should be improved to enhance their performance in having low-risk sexual intercourse and following treatment measures.

Fear is an intensely negative internal state which can cause physiological changes and may thus produce behavioral changes [32]. Our findings showed the role of “fears and concerns” as both facilitators of and barriers to drug use and treatment adherence.

Fear and concern about drug side effects, lack of recovery, examination, COVID-19, and the fear associated with medical treatment was discussed by many patients. Fear can be one of the main reasons for treatment failure and recurrence of infection. Many participants were less willing to take their medication accurately or completely because of fear of side effects, and as a result, the infection did not improve and may have recurred. The results of our study showed that fear of drug side effects influences medication adherence, which is consistent with the results of Watkins et al. [33] and Mostafavi et al. [18]. Fear of illness can be considered a natural response to a dangerous situation, and in some situations, it may be beneficial for the individual to minimize high-risk behaviors, and it is necessary to encourage the patient to adhere to treatment and proceed with the treatment process. Previous studies have shown that fear sometimes causes a person to avoid high-risk behaviors and situations, thereby reducing the chances of getting an infection [34, 35].

Consultation with a specialist and pelvic examinations of women should be done regularly from the age of 21 [36]. However, this is an unpleasant experience for most women with a sense of fear and anxiety. Given that routine pelvic exams are important for preventive health measures, and early detection of some infections, overcoming this fear can lead to negative effects on women's health because of avoiding examination [37]. In cases where the patient does not allow the doctor to examine them, the doctor may prescribe a drug without vaginal examination and only based on the patient's history, which can be a logical reason for treatment failure.

Physicians should adopt stress reduction strategies and pain relief techniques during the examination. These include choosing the right size of the speculum, especially for women who experience pain during vaginal examinations and reassuring the patient, for example, by allowing a companion to be present in the examination room providing support so that the patient can manage her concern and anxiety. Also, considering that in some cultures, Iranian included, losing virginity before marriage is a taboo, and any infection or vaginal disease is stressful for virgins and their families. In many cases, the treatment process starts without a thorough examination and is only based on the patient's history, which can make the disease worse.

With the onset of the COVID-19 epidemic, government restrictions, including the closure of clinics or reduced office hours, were imposed to minimize the spread of the virus. The steady increase in the number of deaths due to COVID-19 and the determination of governments in imposing restrictions led to patients’ fear of going to a specialist office for seeking treatment. A study by Casey et al. showed an unprecedented decline in sexually transmitted disease testing due to restrictions on the COVID-19 epidemic in the United States. Its findings are a warning sign of the potential side effects of sexual and reproductive health [38]. For many of our study participants, saving their lives amidst the COVID-19 pandemic was their top priority, so they tried to treat the infection by self-medication and without consulting a physician, which in most cases led to treatment failure or infection recurrence.

## Strengths and limitations of this study

In this study, we specifically sought to identify factors related to treatment-seeking behaviors in women with VVC which are usually hidden in quantitative studies and have not yet been addressed in qualitative studies. In this study, a new combination of beliefs and fears as barriers and facilitators of adherence to treatment among women with VVC has been introduced, which is considered the strong point of this study. The reluctance of some patients to collaborate with researchers for various reasons, such as embarrassment or lack of family cooperation, is a limitation of this study. However, by explaining the objectives of the study to the participants and their families and reassuring them that they would be kept anonymous during the study, this limitation was partially overcome.

The results of this study showed that many of the patients' behaviors are rooted in their beliefs, fears, and concerns. Therefore, the patients must obtain sufficient scientific information about the infection in simple language before any examination or drug prescription to be mentally prepared for the steps from diagnosis to treatment. Such a goal is achieved through a close relationship between the treatment staff and the patient. Intimate communication leads to patients’ trust in the treatment staff, the preference for a doctor's prescription over beliefs, and the eventual overcoming of fear. However, in many cases, treatment is prescribed only based on predetermined treatment protocols. It is recommended that women be educated about the facts and events related to VVC to reduce the influence of patients' beliefs, fears, and concerns on the progress and/or follow-up of the treatment process; the patient must be psychologically ready to accept the treatment process, especially in cases where the treatment is prolonged and the patient is disappointed with the treatment.

## Conclusion

It seems that some women who contract VVC suffer from this disease for a long time and even for years in some cases. The results of this study showed that many of the patients’ behaviors regarding accepting medication, seeing a doctor, not withdrawing from medication, or allowing a vaginal examination by a specialist are all rooted in the patient's beliefs, fears, and concerns and can lead to treatment adherence or non-adherence. Therefore, it is clear that focusing only on drug treatments and other one-dimensional methods does not necessarily lead to the desired outcome. Rather, it seems necessary to plan and carry out interventions based on the recognized factors in this study. The findings of this study can be used in the design and development of appropriate solutions, treatment guidelines, and policy-making for treatment adherence.

## Data Availability

The data that support the findings of this study are available from the corresponding author upon reasonable request.

## Abbreviations

VVC: Vulvovaginal candidiasis
RVVC: Recurrent vulvovaginal candidiasis
OTC: Over-the-counter
IUD: Intrauterine device
OCD: Obsessive–compulsive disorder

## Footnotes

1 . Having 4 or more episodes per year of VVC was considered RVVC.

## References

[1] Pereira LC, Correia AF, da Silva ZDL, de Resende CN, Brandão F, Almeida RM (2021). Vulvovaginal candidiasis and current perspectives: new risk factors and laboratory diagnosis by using MALDI TOF for identifying species in primary infection and recurrence. Eur J Clin Microbiol Infect Dis.

[2] Yano J, Sobel JD, Nyirjesy P, Sobel R, Williams VL, Yu Q (2019). Current patient perspectives of vulvovaginal candidiasis: incidence, symptoms, management and post-treatment outcomes. BMC Women’s Health.

[3] Shi Y, Zhu Y, Fan S, Liu X, Liang Y, Shan Y (2020). Molecular identification and antifungal susceptibility profile of yeast from vulvovaginal candidiasis. BMC Infect Dis.

[4] Rosati D, Bruno M, Jaeger M, Ten Oever J, Netea MG (2020). Recurrent vulvovaginal candidiasis: an immunological perspective. Microorganisms.

[5] Sobel JD (2016). Recurrent vulvovaginal candidiasis. Am J Obstet Gynecol.

[6] Ghaddar N, Anastasiadis E, Halimeh R, Ghaddar A, Dhar R, AlFouzan W (2020). Prevalence and antifungal susceptibility of *Candida*
*albicans* causing vaginal discharge among pregnant women in Lebanon. BMC Infect Dis.

[7] Jang SJ, Lee K, Kwon B, You HJ, Ko G (2019). Vaginal lactobacilli inhibit growth and hyphae formation of *Candida*
*albicans*. Sci Rep.

[8] Lírio J, Giraldo PC, Amaral RL, Sarmento ACA, Costa APF, Gonçalves AK (2019). Antifungal (oral and vaginal) therapy for recurrent vulvovaginal candidiasis: a systematic review protocol. BMJ Open.

[9] Carr PL, Felsenstein D, Friedman RH (1998). Evaluation and management of vaginitis. J Gen Intern Med.

[10] Rezaei-Matehkolaei A, Shafiei S, Zarei-Mahmoudabadi A (2016). Isolation, molecular identification, and antifungal susceptibility profiles of vaginal isolates of *Candida* species. Iran J Microbiol.

[11] Qin F, Wang Q, Zhang C, Fang C, Zhang L, Chen H (2018). Efficacy of antifungal drugs in the treatment of vulvovaginal candidiasis: a Bayesian network meta-analysis. Infect Drug Resist.

[12] Sobel JD (2014). Factors involved in the patient’s choice of oral or vaginal treatment for vulvovaginal candidiasis. Patient Prefer Adherence.

[13] Kiasat N, Rezaei-Matehkolaei A, Zarei Mahmoudabadi A, Hamidavi Mohamadpour K, Molavi S, Khoshayand N (2019). Prevalence of vulvovaginal candidiasis in Ahvaz, Southwest Iran: a semi-large scale study. Jundishapur J Microbiol.

[14] Kiasat N, Rezaei-Matehkolaei A, Mahmoudabadi AZ (2019). Microsatellite typing and antifungal susceptibility of *Candida*
*glabrata* strains isolated from patients with Candida Vaginitis. Front Microbiol.

[15] Gonçalves B, Ferreira C, Alves CT, Henriques M, Azeredo J, Silva S (2016). Vulvovaginal candidiasis: epidemiology, microbiology and risk factors. Crit Rev Microbiol.

[16] Bradfield Strydom M, Walpola RL, McMillan S, Khan S, Ware RS, Tiralongo E (2022). Lived experience of medical management in recurrent vulvovaginal candidiasis: a qualitative study of an uncertain journey. BMC Womens Health.

[17] Mostafavi-Darani F, Zamani-Alavijeh F, Mahaki B, Salahshouri A (2020). Exploring the barriers of adherence to dietary recommendations among patients with type 2 diabetes: a qualitative study in Iran. Nurs Open.

[18] Mostafavi F, Alavijeh FZ, Salahshouri A, Mahaki B (2021). The psychosocial barriers to medication adherence of patients with type 2 diabetes: a qualitative study. Biopsychosoc Med.

[19] Norenhag J, Du J, Olovsson M, Verstraelen H, Engstrand L, Brusselaers N (2020). The vaginal microbiota, human papillomavirus, and cervical dysplasia: a systematic review and network meta-analysis. BJOG.

[20] Kalia N, Singh J, Kaur M (2020). Microbiota in vaginal health and pathogenesis of recurrent vulvovaginal infections: a critical review. Ann Clin Microbiol Antimicrob.

[21] Grace D, Gaspar M, Paquette R, Rosenes R, Burchell AN, Grennan T (2018). HIV-positive gay men's knowledge and perceptions of Human Papillomavirus (HPV) and HPV vaccination: a qualitative study. PLoS ONE.

[22] Braun V, Clarke V (2006). Using thematic analysis in psychology. Qual Res Psychol.

[23] Rezapour M, Zarghami M, Sheikhmoonesi F (2021). Psychological experience and needs of front-line nurses during COVID-19 outbreak in Iran: a qualitative study. J Mazandaran Univ Med Sci.

[24] Lincoln YS, Guba EG (1985). Naturalistic inquiry.

[25] Tong A, Sainsbury P, Craig J (2007). Consolidated criteria for reporting qualitative research (COREQ): a 32-item checklist for interviews and focus groups. Int J Qual Health Care.

[26] Bilardi J, Walker S, McNair R, Mooney-Somers J, Temple-Smith M, Bellhouse C (2016). Women's management of recurrent bacterial vaginosis and experiences of clinical care: a qualitative study. PLoS ONE.

[27] Brock H, Hany M, Brock H (2021). Obsessive-Compulsive Disorder. StatPearls.

[28] Khosravani V, Samimi Ardestani SM, Sharifi Bastan F, McKay D, Asmundson GJG (2021). The associations of obsessive-compulsive symptom dimensions and general severity with suicidal ideation in patients with obsessive-compulsive disorder: the role of specific stress responses to COVID-19. Clin Psychol Psychother.

[29] Krebs G, Heyman I (2015). Obsessive-compulsive disorder in children and adolescents. Arch Dis Child.

[30] Pérez-Vigil A, Fernández de la Cruz L, Brander G, Isomura K, Gromark C, Mataix-Cols D (2016). The link between autoimmune diseases and obsessive-compulsive and tic disorders: a systematic review. Neurosci Biobehav Rev.

[31] Khalesi ZB, Simbar M, Azin SA (2017). A qualitative study of sexual health education among Iranian engaged couples. Afr Health Sci.

[32] Mobbs D, Adolphs R, Fanselow MS, Barrett LF, LeDoux JE, Ressler K (2019). Viewpoints: approaches to defining and investigating fear. Nat Neurosci.

[33] Anstey Watkins J, Ross JDC, Thandi S, Brittain C, Kai J, Griffiths F (2019). Acceptability of and treatment preferences for recurrent bacterial vaginosis-topical lactic acid gel or oral metronidazole antibiotic: qualitative findings from the VITA trial. PLoS ONE.

[34] Harper CA, Satchell LP, Fido D, Latzman RD (2020). Functional fear predicts public health compliance in the COVID-19 pandemic. Int J Mental Health Addict.

[35] Tzur Bitan D, Grossman-Giron A, Bloch Y, Mayer Y, Shiffman N, Mendlovic S (2020). Fear of COVID-19 scale: psychometric characteristics, reliability, and validity in the Israeli population. Psychiatry Res.

[36] Bialy A, Bialy A (2022). Gynecologic examination. StatPearls.

[37] O'Laughlin DJ, Strelow B, Fellows N, Kelsey E, Peters S, Stevens J (2021). Addressing anxiety and fear during the female pelvic examination. J Prim Care Community Health.

[38] Pinto CN, Niles JK, Kaufman HW, Marlowe EM, Alagia DP, Chi G (2021). Impact of the COVID-19 pandemic on Chlamydia and Gonorrhea screening in the U.S. Am J Prev Med.

